# The impact of pop-up warning messages of losses on expenditure in a simulated game of online roulette: a pilot study

**DOI:** 10.1186/s12889-019-7191-5

**Published:** 2019-06-26

**Authors:** Paul McGivern, Zaheer Hussain, Sigrid Lipka, Edward Stupple

**Affiliations:** 1 York St John University, York, UK; 2 University of Derby, Derby, UK; 3 School of Human Sciences, University of Derby, Kedleston Road, Derby, DE22 1GB UK

**Keywords:** Responsible gambling, Pop-ups, Warning messages, Electronic gaming machines, Harm minimisation

## Abstract

**Background:**

‘Pop-up’ warning messages have potential as a Responsible Gambling tool, but many warning messages in the literature are generic. The present study simulated digital roulette to compare the effectiveness of expenditure-specific, generic and control messages, during online roulette.

**Methods:**

Forty-five casual gamblers participated in a laboratory setting. Gambles were ‘rigged’ such that participants suffered a net loss. Total ‘play money’ wagers from individual bets after the presentation of the messages were measured.

**Results:**

Expenditure-specific warning messages demonstrated significant reductions in wager amounts compared with other message types - Generic (*p* = .035) and Control messages (*p* < .001). No significant differences were found between Generic and Control messages (*p* > .05). Thus expenditure-specific warning messages about current losses were more effective than generic messages for reducing expenditure.

**Conclusions:**

Expenditure-specific warning messages exhibit potential for ameliorating potentially harmful gambling behaviour. Expenditure-specific messages should be tested in a broader range of gambling contexts to examine their generalizability and potential for implementation in the gambling industry.

## Background

Online gambling is the fastest growing form of gambling in the world [1] and roulette is one of the most popular games played by gamblers [2]. Online gambling can be accessed via a range of technologies including mobile devices. Its increasing popularity has contributed to a shift in perspective toward the need for responsible gambling (RG) policies based on informed choice for gamblers [3, 4]. RG tools have been acknowledged as having the potential to prevent casual gamblers from becoming problem gamblers [5]. As a result, RG is now a high priority for digital gambling providers [6] with focus on developing systems that are protective of the majority of the gambling population [7, 8]. The use of ‘pop-up’ warning messages as a device to facilitate safer, more informed gambling has gained notable research focus [9].

To date, many studies examining the effectiveness of warning messages have focused predominantly on the correction of erroneous beliefs, self-appraisal/informative detail, limit-setting and information pertaining to aspects of game-play [8, 10–18] and have produced some promising but varied findings. While messages warning of gamblers fallacies have potential, they have also been shown to have little effect on gambling behaviour [19]. This is further compromised in the game of roulette whereby erroneous beliefs can be triggered in many ways due to the layout of the board and wheel. It is therefore difficult to determine if, and when erroneous beliefs may be occurring [20].

A recent study [21] showed that messages discussing money spent impacted on message recall, and that the optimal position of warning messages was the centre of the screen [22]. While it is broadly acknowledged that in-venue data [21, 22] have greater ecological validity than laboratory-based studies, the authors note the lack of specific behavioural measures and potential inaccuracies of self-report in such studies. Further potential confounds relate to differences in gaming machines. A laboratory-based study such as the current one may contribute towards clarifying the effectiveness of differing warning message content.

Of late, focus has shifted toward the use of personalised feedback as a strategy to combat risky gambling behaviour, and it has been highlighted as an important future avenue for gambling research [23]. This is supported by ecological studies [23] and broader research showing the increased impact of personally tailored information [24]. Such strategies also lend themselves to feedback systems, such as limit-setting devices (combined with the use of warning messages when self-selected time and monetary limits are reached) that have been implemented within many gambling websites. The usefulness of personalised feedback has been explored in several studies [23, 14, 25, 8, 26]. Recently, [27] a voluntary progressive warning message system was also tested for those opting to set pre-determined expenditure limits. The sample (which comprised mostly of casual gamblers) received expenditure-related warning messages when 50 and 90% of losses had occurred (e.g. “You have now spent about half of your money”). Initial reports of the system were positive and further support the application of proportionally based warning messages.

While the potential benefits of limit-setting are self-evident, it has been noted [28] that messages about reaching loss-limits may be emotionally painful (and potentially too late), therefore mandatory warnings prior to reaching this point may be beneficial and assist in quitting gambling sessions. However, some research has shown low usage of such features by the general gambling population [29], highlighting the need to further explore the impact of personalised warning messages about current losses. It may be necessary to make warning messages a mandatory feature of high-risk gambling devices, if research can demonstrate their effectiveness without compromising enjoyment [30]. Indeed, some research indicates that RG strategies largely do not compromise player enjoyment [21, 22]. In Great Britain, consumers only receive mandatory warnings when they have been gambling for 30-min or have spent over £250 [41]. Such limits are impersonal and still arguably too lenient whereby significant losses can be incurred. However, using pop-up messages in an online simulated casino environment are yet to be sufficiently investigated.

The propensity of gamblers to chase losses during gambling sessions has been highlighted [31], further justifying the use of within-session warning messages. Irrational beliefs and illusion of control are also common among regular gamblers [16]. This further supports the need to develop RG tools that are relevant to all gamblers, as previously stated in research [21, 22]; and highlights that within-session warning messages may be effective in combating irrational cognitions that perpetuate gambling behaviour.

Warning messages informing players when they had reached 1000 gambles on a slot-machine effectively encouraged gamblers to stop [32]. A follow-up study showed that adding either normative or self-appraisal style feedback to the same message further increased message effectiveness [10]. Self-appraisal style messages are designed to encourage gamblers to generate their own thoughts about their gambling behaviour (e.g. ‘Have you spent more than you can afford?’) and subsequently to facilitate greater awareness of gambling activity [21]. Although the authors [10] note the limitations of not knowing the levels of gambling pathology of their sample, these findings support the utility of tailored warning message content pertaining to game-play information for within-session gambling. Moreover, normative feedback may be less applicable when playing roulette given the smaller house-edge. Therefore, the number of spins played may not be an appropriate measure of risk in terms of losses, though the use of factually accurate information about in pop-ups may serve to alleviate previously highlighted issues of trust regarding RG information accuracy [40] thus potentially improving their impact. This contrasts with du Preez et al. [43] who indicated that gamblers may doubt the accuracy of pop-up messages. Nonetheless the question of increasing gambler’s engagement with such messages continues to be an open one. In the present paper factually accurate expenditure based pop-up messages were used.

A recent survey among responsible gambling experts, recovered problem gamblers and treatment providers also showed that pop-up messages about specific loss amounts would be useful [33]. These findings align with broader warning message research. In particular, the Elaboration Likelihood Model (ELM) [24] illustrates how warning message impact depends on the degree to which players engage with warning information. People are generally motivated to hold correct attitudes, and increased relevance of warnings can enhance warning message effectiveness by engagement with warning information and positively changing attitudes towards potentially harmful consumer products [24].

Monetary expenditure is a key facet of data-tracking methods used by online gambling operators. Referring to monetary expenditure in warning messages would therefore be both technologically achievable [32] and beneficial to consumers given current research findings as discussed above. Such an approach also partially aligns with the public health approach to harm reduction insofar as it draws on one of the three causes or problematic gambling (the positive use of structural characteristics), which has been argued to be the most important aspect that researchers should focus, particularly for RG tools in Great Britain [42].

The present study aimed to test the efficacy of bespoke pop-up warning messages tailored to individual gambling expenditure, and to examine their impact on subsequent gambling expenditure following exposure to messages. Given the preceding review, it was predicted that expenditure-specific pop-up warning messages (containing specific loss amounts in real time) would have a greater impact on subsequent expenditure compared with generic messages (standard warning message referring to the financial risks of gambling) and control messages (containing no warning content).

## Method

### Participants

Forty-five participants (*n* = 19 males, *n* = 24 females, n = 2 undisclosed) aged 18 years or over took part in the study (participant age was not recorded for this study). The nine-item Problem Gambling Severity Index (PGSI) [34] has been shown to be a robust measure (Cronbach’s Alpha, α = 0.84) of problem gambling [33, 35] and was therefore used to screen-out potential problem gamblers. Using the PGSI, a score of 0 = Non-problem gambling, a score of 1 or 2 = Low level problems with few or no identified negative consequences, scores between 3 and 7 = Moderate level of problems leading to some negative consequences. Scores of 8 or more = Problem gambling. Based on the PGSI, the sample were: Non-problem gamblers (*n* = 31), Low-level problem gamblers (*n* = 9), and Moderate level problem gamblers (*n* = 5). There were no problem gamblers (score of 8 or more on the PGSI) in the study. The overall mean PGSI score was 0.85 (SD = 1.43). Participants responded to each statement of the PGSI framed within the last 12 months.

Participants were university students recruited from the East Midlands and North-East regions of England via convenience sampling. Participants self-identified as gamblers, which was the key criterion for taking part in the study. Participants were informed that after taking part in the study, their remaining simulated game credits would be converted into raffle tickets to win a prize (i.e., a shopping voucher). This method has been previously used to encourage more realistic gambling behaviour in simulation studies [12]. However, to ensure that all participants received a fair chance of winning the raffle, during debriefing participants were informed that this was a deception and that all participants would receive an equal opportunity to win the raffle. The study was approved by the local psychology ethics committee.

### Design

A between-groups design was employed to compare Expenditure-specific warning messages, Generic warning messages, and Control messages to examine differences in total amount wagered during ‘open bets’ (see Procedure for definition of open bets). The aggregated total betting for open bets was the dependent variable.

### Procedure

Participants provided informed consent and completed the PGSI. Those scoring between 0 and 7 on the PGSI then played the game. Participants were informed that they would be playing a simulated game of roulette for approximately 15 min. Participants were randomly allocated to one of the three conditions. Participants started with £1000 simulated credit and were instructed to begin using fixed £50 wager amounts. For most of game-play, participants could only place ‘outside’ bets of £50 (e.g. red; black; odd; even etc.) by selecting tick box options, to control for expenditure fluctuations.

Game-play was fixed such that all players ultimately lost and finished when their credit reached less than £50. The game included fixed wins and losses to control for winning/losing streaks. When participants’ expenditures reached or passed the four fixed loss thresholds of: £750, £500, £250, and £100, warning messages (regardless of message type) were displayed in the centre of the screen and required participants to click an ‘OK’ push button to confirm the message and clear it from the screen. After each message was displayed (as part of the same message), participants were informed that they could then place an ‘open bet’ (multiple choices and stake values) up to £100. The amounts wagered during these four ‘open’ bets were added up to create the dependent variable. No other expenditure measures were collected. The game finished when participants reached their remaining credit limit of £50 and participants were debriefed and thanked for their participation.

### Materials

**Fig. 1:**
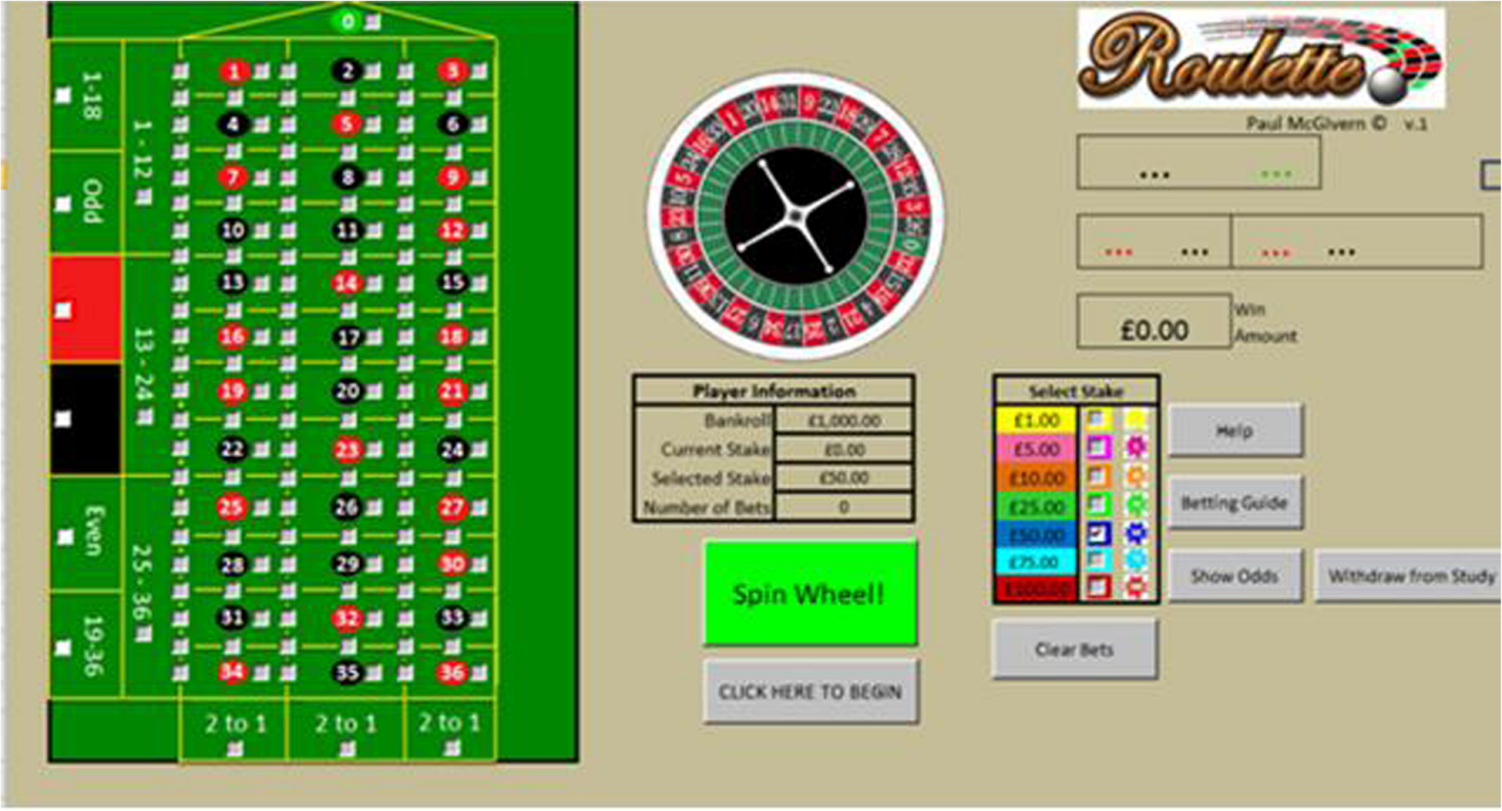
Roulette Screenshot (without example warning message); A screenshot of the software used for the study without any pop-up warning messages displayed

**Fig. 2:**
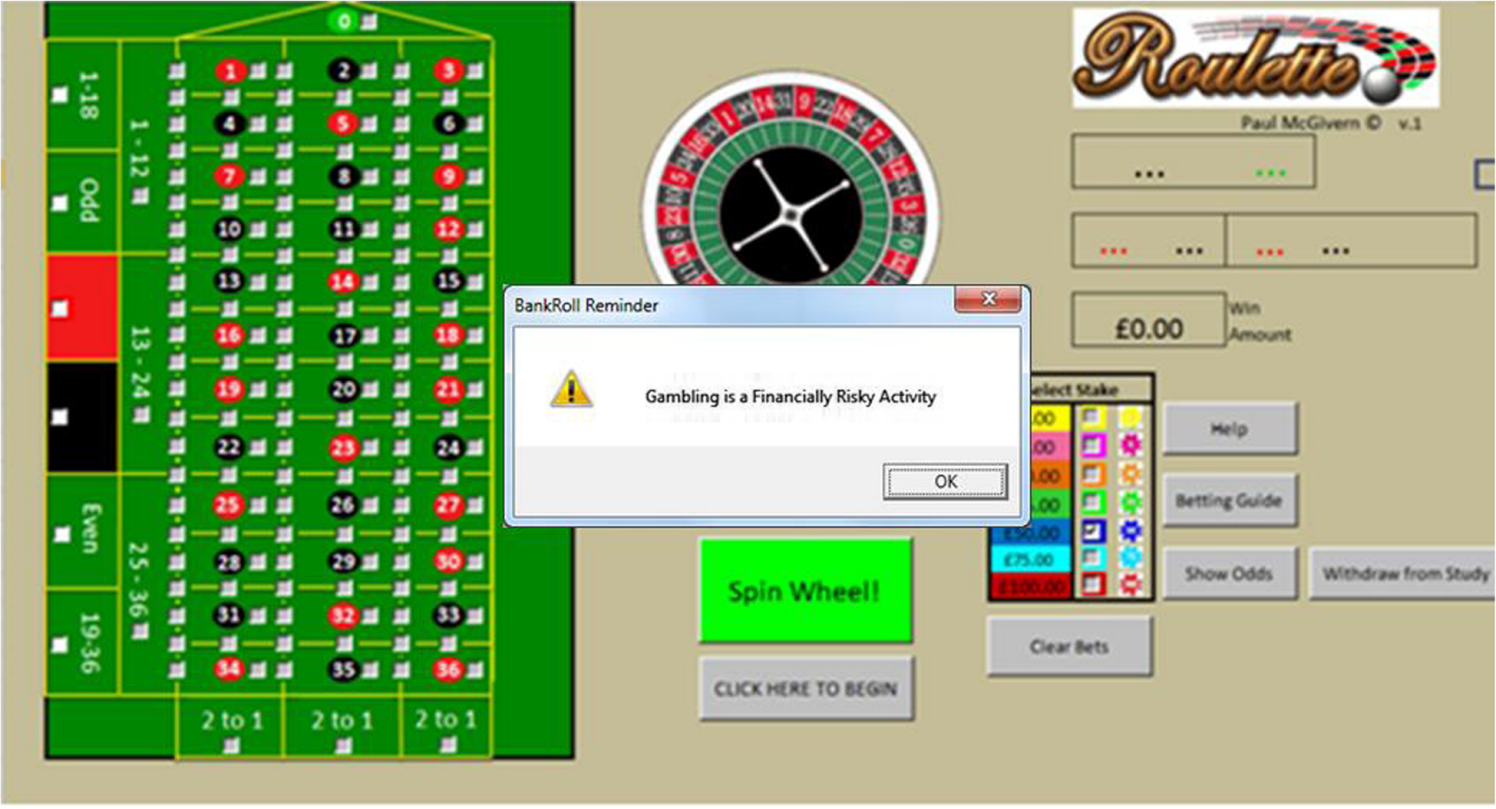
Roulette Screenshot (with example warning message); A screenshot of the software used for the study, which also shows how the screen appeared to participants when pop-up messages appeared on the screen

**Fig. 3:**
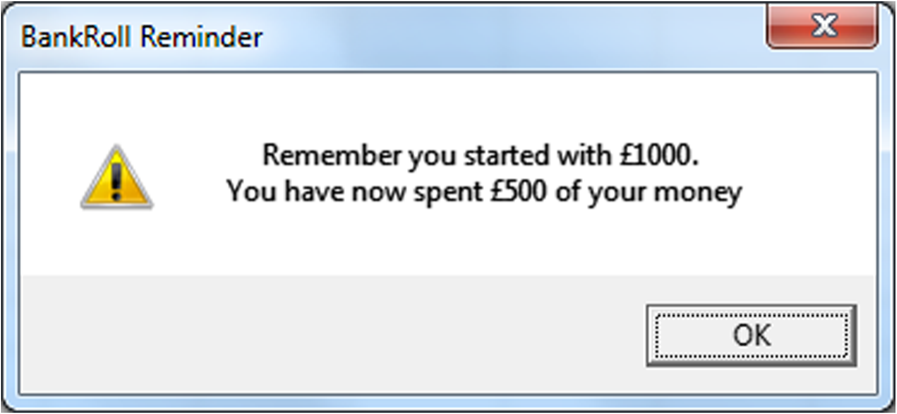
Expenditure-Specific Warning Message; A screenshot example of one of the four expenditure-specific messages displayed to participants in this group. The values displayed in each of the four messages was relevant to their current bankroll position at the time of display

**Fig. 4:**
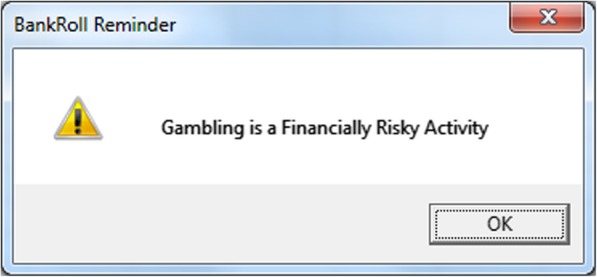
Generic Warning Message; A screenshot example of the generic message displayed to participants in this group

**Fig. 5:**
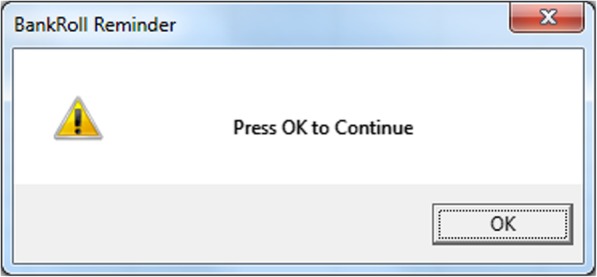
Control Warning Message; A screenshot example of the control message displayed to participants in this group

The first author developed the testing tool using *Microsoft Excel*. The screen displayed a European roulette table and wheel. A stake selection box was available during ‘open bets’ allowing participants to choose between £1, £5, £10, £25, £50, £75, and £100 stake amounts (see Figs. 1 and 2). Player information was displayed next to the roulette wheel and gaming board, tracking expenditure and number of bets placed. Table 1 shows the warnings used in each of the 3 conditions (also see Figs. 3, 4 and 5 for examples of each warning messages).

**Table 1:** Warning Message Information

Group	Message
Expenditure-Specific	“Remember you started with £1000. You have now *spent* £*amount* of your money”
Generic	“Gambling is a Financially Risky Activity”
Control	“Press OK to Continue”

## Results

Table 2 gives means and standard deviations of the dependent variable Total Wager Amount in each of the Message Type conditions.

**Table 2:** Means and Standard Deviations for Total Wager Amount by Message Type Group

	Expenditure Specific		Generic		Control		Total	
	Mean	(SD)	Mean	(SD)	Mean	(SD)	Mean	(SD)
Total Wager Amount	150.07	(92.69)	235.27	(95.41)	305.67	(76.18)	230.33	(107.77)

Differences in Total Wager Amount due to Message Type were examined using a between-subjects ANOVA. There was a significant main effect for Message Type, with a large effect size: F_(2,42)_ = 11.63, *p* < .001, η_p_^2^ = .356. Differences between the three message types were examined using post-hoc Bonferroni adjusted comparisons. These revealed that Expenditure-Specific messages differed significantly from both Generic warning messages (*p* = .035) and Control messages (p < .001), with significantly lower total wager amounts in both cases. Generic messages were not significantly different from control messages (*p* = .105). Follow-up analyses examined the impact of different warning message types on Total Expenditure Amount between males and females for each group; also between and Non-problem and Low-level problem gamblers (in accordance with the PGSI). The analyses found no significant differences in Total Expenditure Amount by message type for Gender or PGSI categorisation (*p* > .05).

## Discussion

This study examined the impact of expenditure-specific messages warning of specific loss amounts in comparison to generic warnings of the financial risks of gambling, and control messages. As predicted, expenditure-specific warning messages were more effective at reducing expenditure and encouraged more responsible gambling compared with the two other message types. The importance of relevant information is further supported by the finding that generic information did not reliably facilitate reduced expenditure, compared with the control information. These findings can be accounted for by the basic principles of the ELM [24] insofar as the expenditure-specific warning messages used in this study can be interpreted as having increased the relevance of accurate information which, according to ELM, results in increased user engagement and subsequently impact on behaviour, in this case, selecting a wager amount. Future studies could further test this interpretation by including a direct measure of user engagement. To better establish the effectiveness of expenditure-specific messages, future studies should also examine their impact in comparison to self-appraisal and informative-style messages and their impact on correcting erroneous beliefs [10, 16, 17, 21, 32].

Expenditure-specific warning messages may complement existing behavioural tracking systems using pop-up warnings that are proportional to user expenditure during play. It has been noted [28] that the industry currently has a preference for self-imposed limit-based approaches due to their reduced interference with game-play. While the expenditure-specific messages in this study used researcher-defined rather than self-imposed limits, the individualisation of information in the messages supports the findings of previous research [e.g. 32, 10].

Ultimately, if gambling operators want to protect their customers without compromising enjoyment, then protective measures such as the pop-ups as used in the current study have potential in facilitating more user-informed gambling in alignment with current initiatives [3]. Furthermore, such an approach partially aligns with public health approaches to harm minimization by making positive use of the structural characteristics of gambling games to promote less risky behaviour [41]. Within-session RG approaches may further assist consumers of gambling products better manage their within-session expenditure. Such an approach may help bring focus to overall within-session expenditure amount. In the present study the pop up messages were designed and tested independently of the gambling industry. In our view these should be complimentary to any broader public health initiative and are consistent with Public Health England’s 2018 strategy [41] calling for the application of behavioural and social sciences to issues of population health.

While the optimisation of content and placement of pop-ups is making progress, more research is required to establish exactly when and how pop-ups should be utilised. Efforts to minimise potential saturation, frustration and/or desensitisation should be considered to prevent jeopardising their application [7, 36]. While the current study supports some research in terms of demonstrating the effectiveness of pop-up messages, further research is required to better understand *when* such messages should be displayed [7], and whether such messages have similar impact on potential problem gamblers.

Some limitations are acknowledged. This study did not examine the impact of players moving to or from winning or losing positions when the messages were displayed, which may have impacted on gambling behaviour. Additionally, the current data only reflect a snapshot of single betting decisions that immediately followed exposure to warnings. This design approach was taken to mitigate against the potential effects of both the low house-edge, and broad variation in gambling options in the game of roulette. However, the drawback to this approach is acknowledged such that the study was only able to capture behaviours immediately following exposure to warning messages prior to restricted game play. Given this, the authors are unable to infer the impact of the warning messages within a less controlled game play environment or on overall gambling behaviour (i.e. whether or not gamblers would have quit the gambling session when reaching zero credits, or if the warning messages had any long-term effects on future gambling). Future studies should attempt capture behaviour across an entire gambling session and measure the impact of expenditure-specific message on the termination of gambling sessions.

Furthermore, the use of simulated credit in a laboratory study [37] with a convenience sample of students warrants caution in generalising results to broader gambling populations [35]. However, establishing effective interventions for encouraging responsible gambling behaviours in younger casual gamblers is an important step in reducing their potential to develop gambling problems [6]. University students form a notable proportion of that group [38] and are in the demographic who both are most likely to develop gambling problems, but are also more likely than other age groups to endorse responsible gambling features/devices/strategies [39]. This is of particular interest and importance, as this demographic represents both a group that warrants further research, and also a group whose potential gambling risk and/or harm could be alleviated and positively addressed, as they are more likely to respond positively to RG features. Finally, the limitation of using PGSI whereby statements were framed over the past 12 months is acknowledged as this did not capture lifetime gambling behaviour. However, given the nature of the study (capturing current behaviours), such an approach was deemed preferable to lifetime gambling behaviours.

With regards to the message content, given the dynamic nature of the expenditure-specific messages in the current study, it could be argued that by comparison to the generic and control warnings it was the dynamic aspect of the message (rather than its contents) which contributed to their effectiveness, and previous research lends some support to this interpretation [15, 21]. However, in order to reduce the potential impact of this, the same phrasing was used in each expenditure-specific warning so that only the loss amounts were different between each of the messages.

## Conclusions

The goal of warning message research in gambling contexts is to reduce or alter risky and/or potentially harmful gambling perceptions and behaviour. The current study showed that expenditure-specific pop-up warning messages reduce expenditure in digital/online roulette. This evidence supports the recommendation to use personally relevant information when attempting to promote more responsible gambling in line with emerging policies that aim to reduce the negative impact of gambling across the general population [7, 8].

## Data Availability

The datasets used and/or analysed during the current study available from the corresponding author on reasonable request.

## Abbreviations

ELM: Elaboration Likelihood Model
PGSI: Problem Gambling Severity Index
RG: Responsible Gambling
